# Integrating multiple oestrogen receptor alpha ChIP studies: overlap with disease susceptibility regions, DNase I hypersensitivity peaks and gene expression

**DOI:** 10.1186/1755-8794-6-45

**Published:** 2013-10-30

**Authors:** Adam E Handel, Geir K Sandve, Giulio Disanto, Lahiru Handunnetthi, Gavin Giovannoni, Sreeram V Ramagopalan

**Affiliations:** 1 Medical Research Council Functional Genomics Unit and Department of Physiology, Anatomy and Genetics, University of Oxford, Oxford OX1 3PT, UK; 2 Blizard Institute, Queen Mary University of London, Barts and The London School of Medicine and Dentistry, London, UK; 3 Department of Informatics, University of Oslo, Blindern, Norway

## Abstract

**Background:**

A wealth of nuclear receptor binding data has been generated by the application of chromatin immunoprecipitation (ChIP) techniques. However, there have been relatively few attempts to apply these datasets to human complex disease or traits.

**Methods:**

We integrated multiple oestrogen receptor alpha (ESR1) ChIP datasets in the Genomic Hyperbrowser. We analysed these datasets for overlap with DNase I hypersensitivity peaks, differentially expressed genes with estradiol treatment and regions near single nucleotide polymorphisms associated with sex-related diseases and traits. We used FIMO to scan ESR1 binding sites for classical ESR1 binding motifs drawn from the JASPAR database.

**Results:**

We found that binding sites present in multiple datasets were enriched for classical ESR1 binding motifs, DNase I hypersensitivity peaks and differentially expressed genes after estradiol treatment compared with those present in only few datasets. There was significant enrichment of ESR1 binding present in multiple datasets near genomic regions associated with breast cancer (7.45-fold, p = 0.001), height (2.45-fold, p = 0.002), multiple sclerosis (5.97-fold, p < 0.0002) and prostate cancer (4.47-fold, p = 0.0008), and suggestive evidence of ESR1 enrichment for regions associated with coronary artery disease, ovarian cancer, Parkinson’s disease, polycystic ovarian syndrome and testicular cancer. Integration of multiple cell line ESR1 ChIP datasets also increases overlap with ESR1 ChIP-seq peaks from primary cancer samples, further supporting this approach as helpful in identifying true positive ESR1 binding sites in cell line systems.

**Conclusions:**

Our study suggests that integration of multiple ChIP datasets can highlight binding sites likely to be of particular biological importance and can provide important insights into understanding human health and disease. However, it also highlights the high number of likely false positive binding sites in ChIP datasets drawn from cell lines and illustrates the importance of considering multiple independent experiments together.

## Background

Many diseases are typified by an unequal prevalence in females and males [1]. This gender disparity is particularly marked for autoimmune diseases, especially multiple sclerosis (MS) where the gender ratio is 2–3:1 [2,3]. Coronary artery disease (CAD) has a lower frequency in premenopausal women and male gender is associated with higher mortality [1,4]. Parkinson’s disease (PD) is more frequent amongst males than females [5]. Clearly certain diseases are either almost or completely gender-specific, such as gynaecological, testicular and prostate cancer.

Much molecular work has focussed upon potential mechanisms underlying gender disparity observed in many of the above diseases. In autoimmunity, oestrogen has been shown to inhibit T-cell expansion and, in MS, alters T-cell proliferation and cytokine secretion in response to neutrally-derived antigens [6-9]. Oestrogen also has been shown to promote neuronal survival in neurodegenerative and neuroinflammatory conditions, including PD and MS [10-12]. The cardioprotective effects of oestrogen are well-described and are thought to be mediated by vasodilatation and decreased atherosclerosis [13]. Oestrogen receptor alpha (ESR1) is required for the vasoprotective effects of oestrogen [14]. Oestrogen replacement therapy has a well-described association with gynaecological malignancy [15]. Differential ESR1 binding is associated with outcome following breast cancer and altered ESR1 expression in ovarian cancer is associated with prognosis [16,17]. A role for oestrogen in testicular and prostate cancer has also been recognised [18-21].

Chromatin immunoprecipitation with chip hybridisation (ChIP-chip) or with massively parallel sequencing (ChIP-seq) has generated a vast amount of data in multiple different cell types regarding ESR1 binding across the genome. Combining ESR1 ChIP-seq datasets have provided insights into enhancer activity [22,23]. There is great potential from combining transcription factor ChIP-seq or epigenomics to provide insights into disease pathophysiology [24]. Previous studies have revealed enrichment for disease-associated variants with markers of open chromatin and disease-relevant transcription factor binding sites [25-28].

We aimed to analyse whether ESR1 binding is enriched in regions associated with diseases and traits that show marked gender disparity and to what extent biologically important information can be obtained by combining ChIP-chip/-seq data obtained by different methodologies. Our study differs from previous attempts to integrate ESR1 ChIP studies by assessing the degree to which useful information can be obtained regardless of specific methodology used. Our hope is that our findings may allow further functional work to focus on candidate variants likely to be important in diseases or traits showing sexual disparity. It is also likely that many ESR1 ChIP peaks are false positives and we aimed to assess whether overlap between different ChIP datasets may enable identification of peaks that are more likely to be true positive binding sites [29].

## Methods

### ChIP-chip/-seq data

We included 15 ESR1 ChIP-chip/-seq datasets where cells had been stimulated with estradiol prior to ChIP from Cistrome and Medline [30-42]. We also included ESR1 ChIP-seq peaks common to all primary cancer cell samples from breast cancer patients [16]. The characteristics of these studies are shown in Additional file 1: Table S1.

### Motif analysis

*De novo* motif discovery was undertaken using MEME-ChIP searching the central 200 bases within each binding interval for either one or no motif per site between 6 and 30 bases in length [43]. STAMP was used to analyse motifs generated by MEME-ChIP for similarity between different datasets using the settings “Metric = PCC, Alignment = SWU, Gap-open = 1000, Gap-extend = 1000,-nooverlapalign Multiple Alignment = IR, Tree = UPGMA, Matching against: user-defined” [44]. The central 200 basepairs of each binding site and the region within 20 bases of SNPs either associated with disease or in linkage disequilibrium with r^2^ ≤ 0.8 were scanned for JASPAR 2009 ESR1 binding motif (MA0112.2) occurrences with p ≤ 0.0001 using FIMO [45].

### ESR1 enrichment within disease/trait-associated genomic regions and hierarchal clustering analysis

The Genomic HyperBrowser was used to determine overlap and hierarchical clustering between different datasets [46,47]. Tracks comprising different genomic features are annotated as segments, which are different stretches of specific chromosomes, or points, which are basepair locations on specific chromosomes. We defined disease-associated genomic regions as those within 100 kb of SNPs in the Genome Wide Association Study Catalogue (downloaded on 30/03/2013) with a p-value ≤1×10^-7^ for pre-defined conditions/traits with known gender disparity (androgen levels, estradiol levels, breast cancer, coronary heart disease, height, male baldness, menopause, menarche, migraine, multiple sclerosis, ovarian cancer, Parkinson’s disease, polycystic ovarian syndrome, prostate cancer, sex hormone binding globulin levels and testicular cancer) in a similar manner to a previous study [48,49]. Overlap was determined using segment-segment analysis with 10,000 Monte-Carlo randomisations maintaining the empiric distribution of segment and inter-segment lengths, but randomising positions. We generated an intensity track based on the proximity of all ESR1 binding sites to the nearest gene. ESR1 binding intervals were represented as points (midpoints of ESR1 binding peaks) and a point-segment analysis using 1,000 Monte-Carlo randomisations with points sampled according to the intensity track, were used to compute p-values (disease/trait-associated regions were represented as segments as before). Hierarchical clustering analysis was performed in the Genomic HyperBrowser by obtaining pairwise overlap-enrichment values for each of the samples and computing distance between samples as the inverse of these values. DNase I hypersensitivity peaks were obtained from UCSC Table Browser [50] and GRO-seq data on differentially expressed genes with estradiol treatment (q ≤ 0.001 for at least one timepoint) from [51]. The midpoint of ESR1 binding sites were classified as falling within exons, introns, UTR, up-/down-/up&down-stream (5 kb) or intergenic regions relative to RefSeq genes.

## Results

### ESR1 binding sites in different datasets

**Figure 1:**
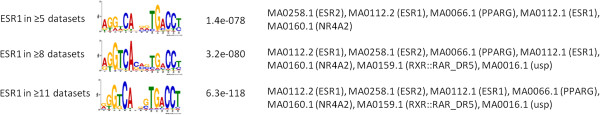
**MEME-identified motifs within ESR1 binding sites in common between datasets.** E-values are shown for each motif along with TOMTOM similarity to known motifs (JASPAR (upper case) and uniprobe mouse (lower case) with E-value <10).

**Figure 2:**
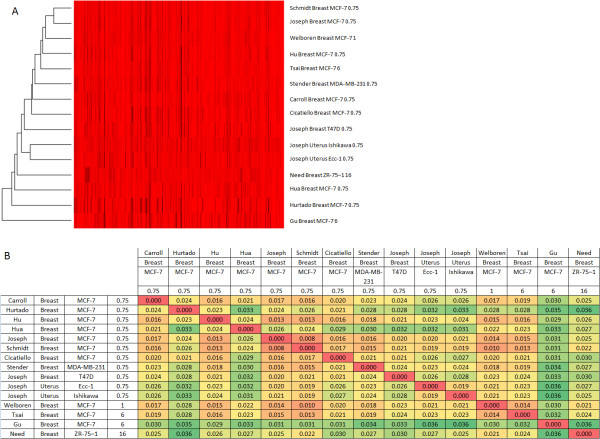
**Hierarchical clustering of ESR1 binding sites between different datasets. (A)** A dendrogram and heatmap of genomic binding illustrating clustering between different datasets. **(B)** A heat map showing relative distances between pairs of datasets (red = 0.000 to dark green = 0.036; units are the inverse of pairwise overlap between ChIP datasets). Study details show the first author, tissue type, cell type and length of estradiol treatment.

The characteristics of each study are shown in Additional file 1: Table S1. Overall, between the 15 datasets, 127,193 ESR1 binding sites were identified. 89,964 (70.7%) were unique to a single dataset, 19,833 (15.6%) were common to at least 3 datasets, 8,390 (6.6%) to at least 5 datasets, 2,880 (2.3%) to at least 8 datasets and 897 (0.7%) to at least 11 datasets. Even when restricting analysis to only those studies conducted in MCF-7 cells exposed to estradiol for 45 minutes, 69.1% of binding sites were unique to a single dataset. Certain regions of the genome are known to generate false positive ChIP-seq peaks, however, only a minority of ESR1 binding sites are located within these regions, and none which are common to highly shared binding sites (Additional file 1: Table S2) [52]. The genomic distribution of ESR1 binding sites was similar regardless of the shared number of datasets (Additional file 1: Table S3). In 12 datasets, the top motif identified by MEME was highly similar to the consensus ESR1 motif on JASPAR 2009 [53] (Additional file 2: Figure S1). The motifs in those 12 showed significant similarity with one another when analysed in STAMP, as did motifs detected in the remaining three, although these were of uncertain biological significance. In all datasets, DREME identified motifs showing significant similarity to ESR1 motifs (Additional file 3: Figure S2). Motif analysis conducted on intervals shared between datasets is shown in Figure 1. In each case, the top motif identified showed significant similarity to the consensus ESR1 motif. The presence of a JASPAR 2009 consensus ESR1 motif (at p ≤ 0.0001) was significantly correlated with the number of shared datasets for each binding site from 13.7% of binding sites unique to a single dataset to 46.3% of those shared between all 15 datasets (r^2^ = 0.71, p = 0.0008, Additional file 1: Table S4). We conducted MEME and DREME analysis in those ESR1 ChIP-seq peaks shared between at least 5 datasets that were found to lack a classical ESR1 recognition motif. MEME was unable to identify any ESR1-like motifs but did identify an SP1-like motif (Additional file 4: Figure S3). The top DREME motif was a FOXA1 binding motif and the second was a partial ESR1/2 motif, suggesting that in some binding intervals lacking the classical ESR1 recognition motif, ESR1 may interact with a degenerate motif. FOXA1 has been described as a binding partner of ESR1 in previous studies and is involved in chromatin interactions [54]. Interestingly MEME and DREME did not identify any FOXA1-like motif in ChIP-seq peaks containing ESR1 classical motifs but did identify similar SP1-like motifs. Hierarchical clustering analysis showed that the ESR1 ChIP datasets conducted in cells derived from uterine tissue cluster together but there was no other obvious clustering based on either the type of breast cancer cells or the length of estradiol treatment used (Figure 2). Only two studies explicitly detailed the use of more than one biological replicates per treatment condition (Carroll *et al.*[30] and Hurtado *et al.*[31]) and there was a trend towards a lower proportion of ESR1 ChIP-seq peaks being unique to a single dataset in these (r^2^ = 0.19, p = 0.054) and a higher proportion of highly shared peaks, which reached significance in those shared between 8 and 10 datasets (8 datasets: r^2^ = 0.27, p = 0.02; 9 datasets: r^2^ = 0.25, p = 0.03 and 10 datasets r^2^ = 0.21, p = 0.04).

### ESR1 enrichment within disease/trait-associated regions

There was highly significant enrichment of ESR1 binding sites within genomic regions associated with breast cancer, height, MS and prostate cancer, which was consistent over multiple datasets and increased in magnitude for binding sites shared between multiple datasets (Additional file 1: Table S5, Table 1). There was suggestive enrichment for CAD, ovarian cancer, Parkinson’s disease, polycystic ovarian syndrome and testicular cancer, although these were not consistent findings across all datasets. ESR1 binding site enrichment within disease/trait-associated regions remained significant when controlling for the position of genes. We also assessed disease/trait overlap using the central 200 bases of each binding site to control for differing size of binding sites and found very similar results (Additional file 1: Table S6). 645 SNPs either directly associated with diseases/traits or in strong linkage disequilibrium (r^2^ ≥ 0.8) were located within ESR1 binding sites but only 12 of these directly disrupted a classical ESR1 recognition motif (Additional file 1: Table S7).

**Table 1:** Enrichment of ESR1 binding sites within genomic regions associated with diseases or traits from GWAS

**GWAS disease/trait**	**All ESR1**		**ESR1 in ≥3 datasets**		**ESR1 in ≥5 datasets**		**ESR1 in ≥8 datasets**		**ESR1 in ≥11 datasets**	
	O/E	p	O/E	p	O/E	p	O/E	p	O/E	p
Androgen levels	1.02	0.8857	0.75	0.8063	0.67	0.9093	1.60	0.4880	4.47	0.1062
Estradiol levels	1.67	0.8659	1.21	0.7437	1.06	0.8905	1.82	0.6311	0.00	1.0000
**Breast cancer**	**2.09**	**0.0002**	**3.21**	**0.0004**	**3.92**	**0.0012**	**5.98**	**0.0038**	**7.45**	**0.0012**
		**(0.002)**		**(0.002)**		**(0.002)**		**(0.002)**		**(0.002)**
**Coronary artery disease**	**1.43**	**0.0002**	**1.69**	**0.0068**	**1.74**	**0.0270**	1.90	0.0704	0.49	0.4322
		**(0.002)**		**(0.002)**		**(0.005)**				
**Height**	**1.55**	**0.0002**	**1.77**	**0.0002**	**1.87**	**0.0004**	**2.36**	**0.0012**	**2.45**	**0.0018**
		**(0.002)**		**(0.002)**		**(0.002)**		**(0.004)**		**(0.004)**
Male baldness	0.75	0.1652	0.42	0.1840	0.37	0.3384	0.41	0.7137	1.16	0.5043
Menopause	1.05	0.8431	0.89	0.7393	0.67	0.4668	0.59	0.5813	0.64	0.9471
Menarche	1.11	0.4504	1.20	0.5341	1.30	0.4410	1.60	0.2680	1.07	0.9173
Migraine	1.29	0.2036	1.55	0.2554	2.42	0.0618	1.32	0.6031	0.00	1.0000
**Multiple sclerosis**	**2.98**	**0.0002**	**2.98**	**0.0002**	**3.40**	**0.0002**	**4.22**	**0.0008**	**5.97**	**0.0002**
		**(0.002)**		**(0.002)**		**(0.002)**		**(0.002)**		**(0.047)**
**Ovarian cancer**	**1.79**	**0.0134**	1.93	0.2676	2.64	0.1596	3.61	0.1564	0.00	1.0000
		**(0.002)**								
**Parkinson’s disease**	**1.54**	**0.0228**	1.65	0.2158	1.62	0.3910	1.17	0.9067	1.35	0.9077
		**(0.002)**								
**Polycystic ovarian syndrome**	**2.42**	**0.0002**	**3.60**	**0.0038**	2.20	0.3386	1.79	0.6467	3.21	0.3286
		**(0.002)**		**(0.022)**						
**Prostate cancer**	**1.82**	**0.0002**	**2.40**	**0.0002**	**2.62**	**0.0006**	**2.76**	**0.0168**	**4.47**	**0.0008**
		**(0.002)**		**(0.002)**		**(0.002)**		**(0.002)**		**(0.002)**
Sex hormone binding globulin levels	1.09	0.8553	0.70	0.4008	0.46	0.2856	0.29	0.3958	0.00	1.0000
**Testicular cancer**	**1.52**	**0.0056**	1.36	0.3218	0.57	0.7883	0.92	0.7523	2.59	0.1498
		**(0.002)**								

Enrichment is shown by differing numbers of shared datasets. Significant enrichment is highlighted in bold and the p-value corrected for genomic location of genes is shown in parenthesis after nominally significant enrichments. O/E = observed/expected ratio.

### DNase I hypersensitivity peaks, gene expression and ESR1 binding

**Figure 3:**
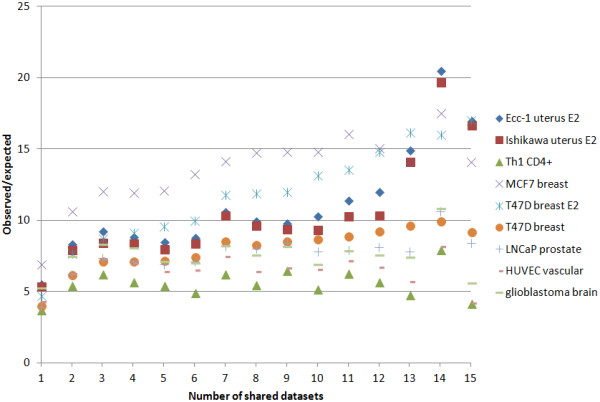
**Enrichment of ESR1 binding sites within DNase I hypersensitivity peaks.** Different cell types are shown as different series where E2 = estradiol-treated.

ESR1 binding sites were significantly enriched for DNaseI hypersensitivity peaks drawn from multiple cell types (Ecc-1, Ishikawa, MDF-7, T47D, LNCaP, HUVEC, glioma and Th1 CD4+, Additional file 1: Table S8). Interestingly, for all apart from Th1 CD4+ cells (r^2^ = 0.05, p = 0.41), HUVEC (r^2^ = 0.002, p = 0.86) and glioblastoma (r^2^ = 0.05, p = 0.41), there was a significant correlation between the number of shared datasets at each ESR1 binding site and the enrichment for DNase I hypersensitivity peaks (Ecc-1 r^2^ = 0.75, p < 0.0001; Ishikawa r^2^ = 0.70, p < 0.0001; MCF7 r^2^ = 0.67, p = 0.0002; T47D plus estradiol r^2^ = 0.97, p < 0.0001; T47D r^2^ = 0.82, p < 0.0001; and LNCaP r^2^ = 0.61, p = 0.0006; Figure 3). This relationship was maintained when ESR1 binding sites were trimmed to the central 200 basepairs to control for differing binding site lengths. ESR1 binding in regions associated with MS were highly enriched for Th1 CD4+ DNase I hypersensitivity peaks (7.41-fold, p < 0.0002) and those in regions associated with prostate cancer for LNCaP DNase I hypersensitivity peaks (9.39-fold, p < 0.0002).

There was significant enrichment of ESR1 binding sites with 5 kb of genes differentially expressed following estradiol treatment (Additional file 1: Table S8) [51]. The degree of enrichment was highly correlated with the number of shared datasets for each ESR1 binding site (r^2^ = 0.89, p < 0.0001), which again was unaffected by restricting analysis to only the central 200 basepairs of each binding interval (r^2^ = 0.94, p < 0.0001).

### Biological significance of motif/DNase I hypersensitivity

We divided ESR1 binding sites into those with and without ESR1 classical motifs. There was some evidence that binding sites with a motif present were more frequently located near disease/trait-associated regions than those without in (breast cancer, coronary artery disease, height, Parkinson’s disease and prostate cancer) but this was not true for all diseases/traits (Additional file 1: Table S9). Motif-containing binding sites were consistently more enriched for DNase I hypersensitivity peaks in all cell types and within 5 kb of genes expressed differently with estradiol treatment than those without motifs.

**Figure 4:**
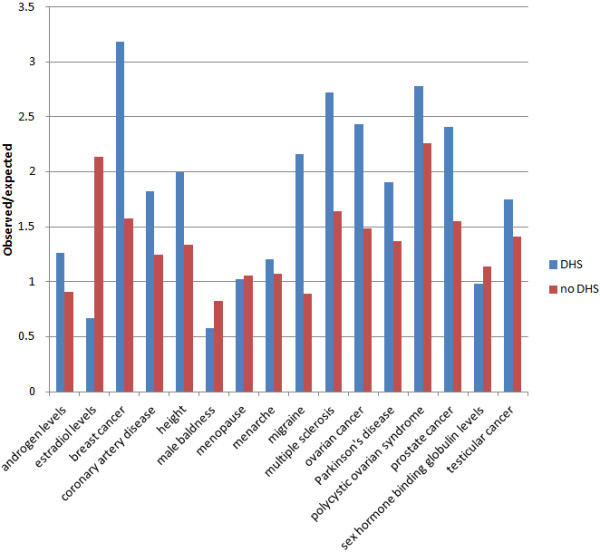
**Enrichment of ESR1 binding sites overlapping and not overlapping DNase I hypersensitivity peaks within disease/trait-associated regions.** ESR1 binding sites are divided into those overlapping and not overlapping DNase I hypersensitivity peaks in the MCF7 breast cancer cell line.

We also separated ESR1 binding sites into those overlapping and not overlapping DNase I hypersensitivity peaks. Disease/trait-associated regions were highly enriched for DNase I hypersensitivity peaks (Figure 4; Additional file 1: Table S9). Motif analysis on ESR1 binding sites overlapping and not overlapping DNase I hypersensitivity peaks common to at least 5 datasets showed ESR1 recognition motifs as the top motif in both cases. Similarly, in both cases motifs similar to FOXA1 were identified.

### Primary cancer ESR1 ChIP-seq

It is possible that the ESR1 ChIP overlap findings are biased by being conducted in cell lines. We analysed ESR1 ChIP-seq data drawn from primary breast cancer samples in relation to GWAS disease/trait regions, DNase I hypersensitivity peaks and estradiol-induced gene expression (Additional file 1: Table S10) [16]. There was significant enrichment of ESR1 binding in GWAS regions associated with androgen levels (11.76-fold, p = 0.02) and breast cancer (11.76-fold, p = 0.0008) with trends for several other conditions/traits. There was highly significant enrichment of ESR1 binding within DNase I hypersensitivity peaks from all cell types studied and near genes differentially expressed with estradiol treatment.

There was a strong correlation between ESR1 ChIP-seq peaks in primary cancer samples and the number of shared datasets for each ESR1 binding site in cell lines (r^2^ = 0.74, p < 0.0001), which was preserved when restricting analysis to only the central 200 basepairs of each ESR1 binding site (r^2^ = 0.73, p < 0.0001).

## Discussion

There is huge variation between individual ESR1 ChIP experiments with most binding sites unique to a single experiment. This may stem from the frequent use of immortalised cell lines, which are known to accrue mutations on prolonged culture and thus may generate a large number of false positive binding sites [55,56]. ESR1 binding sites shared between multiple experiments are likely to be more important to regulatory activity given the higher enrichment of DNase I hypersensitivity peaks, differentially expressed genes and ESR1 binding motifs at highly shared sites compared with sites shared only between few experiments. This is supported by increased enrichment of ESR1 binding sites near regions associated with diseases/traits when those binding sites contain motifs or DNase I hypersensitivity peaks. This suggests that integrating multiple different published ChIP datasets is important in mapping the most important binding sites for transcription factors and can provide valuable biological insights even if the precise methodologies used in each experiment differ [22]. Our analysis thus suggests that integration of multiple ChIP datasets, especially in cell lines, is important to distinguish true positive from false positive ChIP peaks. This is supported by far higher overlap of ESR1 ChIP-seq peaks in primary cancer cells with cell line ChIP peaks found in multiple datasets. Our results also emphasise the importance of analysing biological replicates. However, one key limitation of ChIP-seq from primary cell lines is that, due to the more differentiated nature of primary cells, nuclear factor binding is less likely to be informative of overlap with susceptibility regions in diseases not primarily affecting that tissue type. Further work will be needed to reveal whether similar relationships between biological importance and preservation of ChIP peaks in multiple datasets exists for other nuclear receptors and transcription factors. This also underlines the importance of uploading raw data on all ChIP experiments so that datasets can be directly compared by calling peaks in the same manner.

Interestingly, ESR1 ChIP peaks identified in breast or uterine cell lines also show significant enrichment for DNase I hypersensitivity peaks from other cell lines, which suggests that functional annotation of the genome may be able to cast some light even on biological pathways in cell lines far removed from the ChIP-seq material. This makes the ENCODE approach a very powerful one, since functional genomics data could potentially be used to generate powerful hypotheses about biological systems removed from the particular one used in an individual experiment [57].

We found that ESR1 binding sites were strongly enriched near regions associated with susceptibility to breast cancer, height, MS and prostate cancer, suggesting that ESR1 may contribute to the functional genomics of these diseases. We have shown that susceptibility SNPs frequently fall beneath ESR1 ChIP peaks and thus suggest a possible functional basis for several GWAS susceptibility SNPs. Some of these were supported by ESR1 ChIP-seq peaks derived from primary cancer samples but this is likely limited by the small number of binding sites in common between samples and the relatively differentiated nature of the chromatin architecture compared with cell lines. Further work should concentrate on integrating expression data with known ESR1 ChIP-seq peaks in order to dissect out the precise details of this interaction between ESR1 binding and disease susceptibility. Focussing on the variants highlighted in our analysis for further functional studies may provide direct evidence of disease susceptibility variants affecting ESR1 binding. ESR1 is an attractive candidate, the binding of which may underlie several diseases showing marked gender disparity. This may ultimately permit the identification of novel biochemical pathways that provide new therapeutic targets [58].

## Conclusions

We have shown that integration of ChIP datasets drawn from multiple different cell lines is a powerful technique to screen out false positive nuclear factor binding sites. Moreover, ESR1 binding sites present in multiple experiments were enriched for ESR1 ChIP-seq peaks from primary cancer samples, DNase I hypersensitivity regions, genes differentially expressed after exposure to estradiol, and regions associated with diseases and traits characterised by sexual disparity. Future work should attempt to use primary cells whenever possible and should focus on potential functional variants that may be linked with human phenotypes identified in this study.

## Competing interests

The authors declare that they have no competing interests.

## Authors’ contributions

AEH and SVR conceived and designed the study. AEH and GKS performed analysis of the data. AEH, GKS, GD, LH, GG and SVR wrote the manuscript. All authors read and approved the final manuscript.

## Pre-publication history

The pre-publication history for this paper can be accessed here:

http://www.biomedcentral.com/1755-8794/6/45/prepub

## Supplementary Material

Additional file 1 **We have included the following additional files.Table S1.** Characteristics of included studies; **Table S2.** ESR1 binding sites overlapping with blacklisted regions; **Table S3.** Genomic location of ESR1 binding sites relative to the number of shared datasets; **Table S4.** Frequency of ESR1 binding sites possessing at least one JASPAR ESR1 motif (MA0112.2); **Table S5.** ESR1 enrichment within genomic regions associated with diseases/traits (O/E = observed/expected); **Table S6.** ESR1 enrichment within genomic regions associated with diseases/traits for central 200 bps of each interval (O/E = observed/expected); **Table S7.** GWAS SNPs or those in LD (r^2 ≥ 0.8) within ESR1 binding sites; **Table S8.** ESR1 enrichment within DNase I hypersensitivity peaks and estradiol differentially expressed genes (n.d. = not done); **Table S9.** ESR1 with and without motifs or DNase I hypersensitivity peaks enrichment within genomic regions associated with diseases/traits for central 200 bps of each interval (O/E = observed/expected; DHS = DNase I hypersensitivity peaks); and **Table S10.** Overlap of ESR1 ChIP-seq binding sites from primary cancer samples (O/E = observed/expected). Click here for file

Additional file 2: Figure S1 MEME-identified motifs within ESR1 binding sites for individual datasets. E-values are shown for each motif along with TOMTOM similarity to known motifs (JASPAR (upper case) and uniprobe mouse (lower case) with E-value <10). Study details show the first author, tissue type, cell type and length of estradiol treatment. Click here for file

Additional file 3: Figure S2 ESR1-like DREME-identified motifs within ESR1 binding sites for individual datasets. E-values are shown for each motif along with TOMTOM similarity to known motifs (JASPAR (upper case) and uniprobe mouse (lower case) with E-value <10). The motif shown is the top motif by E-value for all except Carroll *et al.* (second top) and Need *et al.* (third top). Study details show the first author, tissue type, cell type and length of estradiol treatment. Click here for file

Additional file 4: Figure S3 MEME- and DREME- identified motifs within ESR1 binding sites without classical ESR1 recognition motifs. E-values are shown for each motif along with TOMTOM similarity to known motifs (JASPAR (upper case) and uniprobe mouse (lower case) with E-value <10). Click here for file
