# Supplementary figures and images for: Integrating multiple oestrogen receptor alpha ChIP studies: overlap with disease susceptibility regions, DNase I hypersensitivity peaks and gene expression
Source: BMC Med Genomics. 2013 Oct 30;6:45. doi: 10.1186/1755-8794-6-45 (PMC4228442; doi:10.1186/1755-8794-6-45)

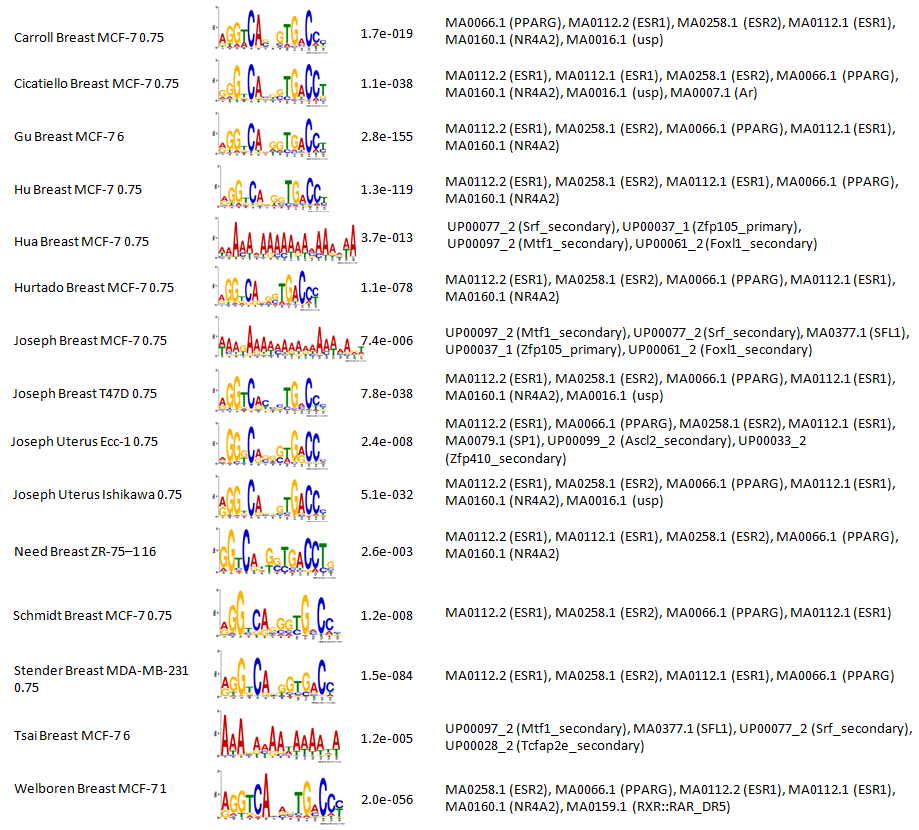

Supplement: Additional file 2: Figure S1 — MEME-identified motifs within ESR1 binding sites for individual datasets. E-values are shown for each motif along with TOMTOM similarity to known motifs (JASPAR (upper case) and uniprobe mouse (lower case) with E-value <10). Study details show the first author, tissue type, cell type and length of estradiol treatment. [file 1755-8794-6-45-S2.tiff]

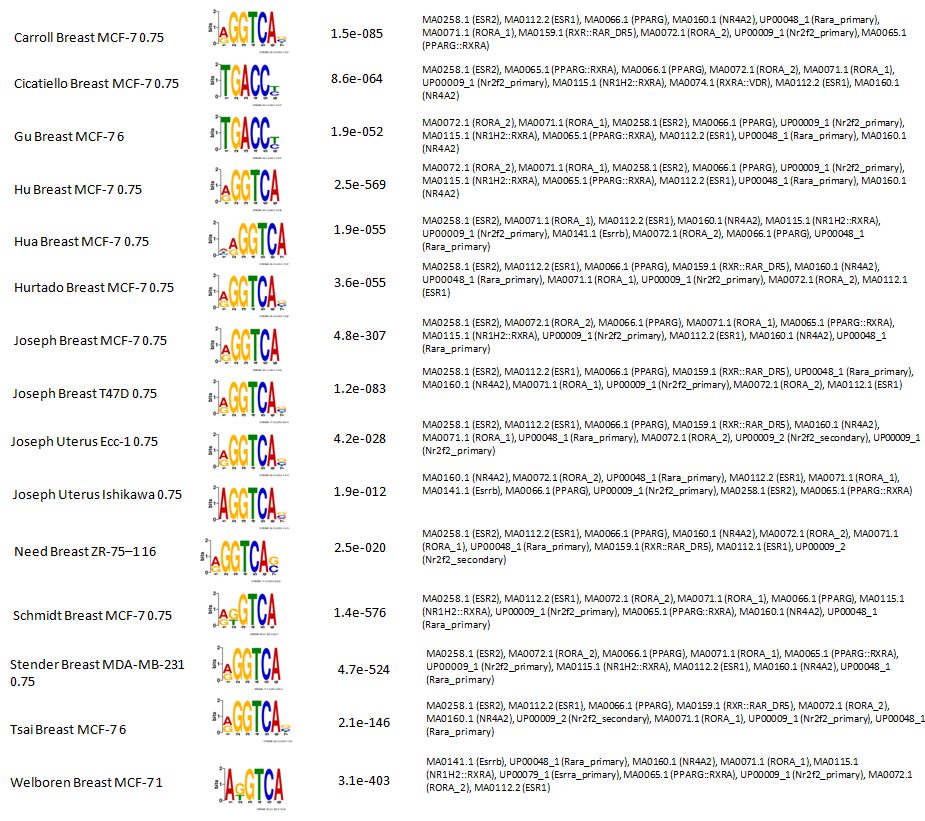

Supplement: Additional file 3: Figure S2 — ESR1-like DREME-identified motifs within ESR1 binding sites for individual datasets. E-values are shown for each motif along with TOMTOM similarity to known motifs (JASPAR (upper case) and uniprobe mouse (lower case) with E-value <10). The motif shown is the top motif by E-value for all except Carroll et al. (second top) and Need et al. (third top). Study details show the first author, tissue type, cell type and length of estradiol treatment. [file 1755-8794-6-45-S3.tiff]

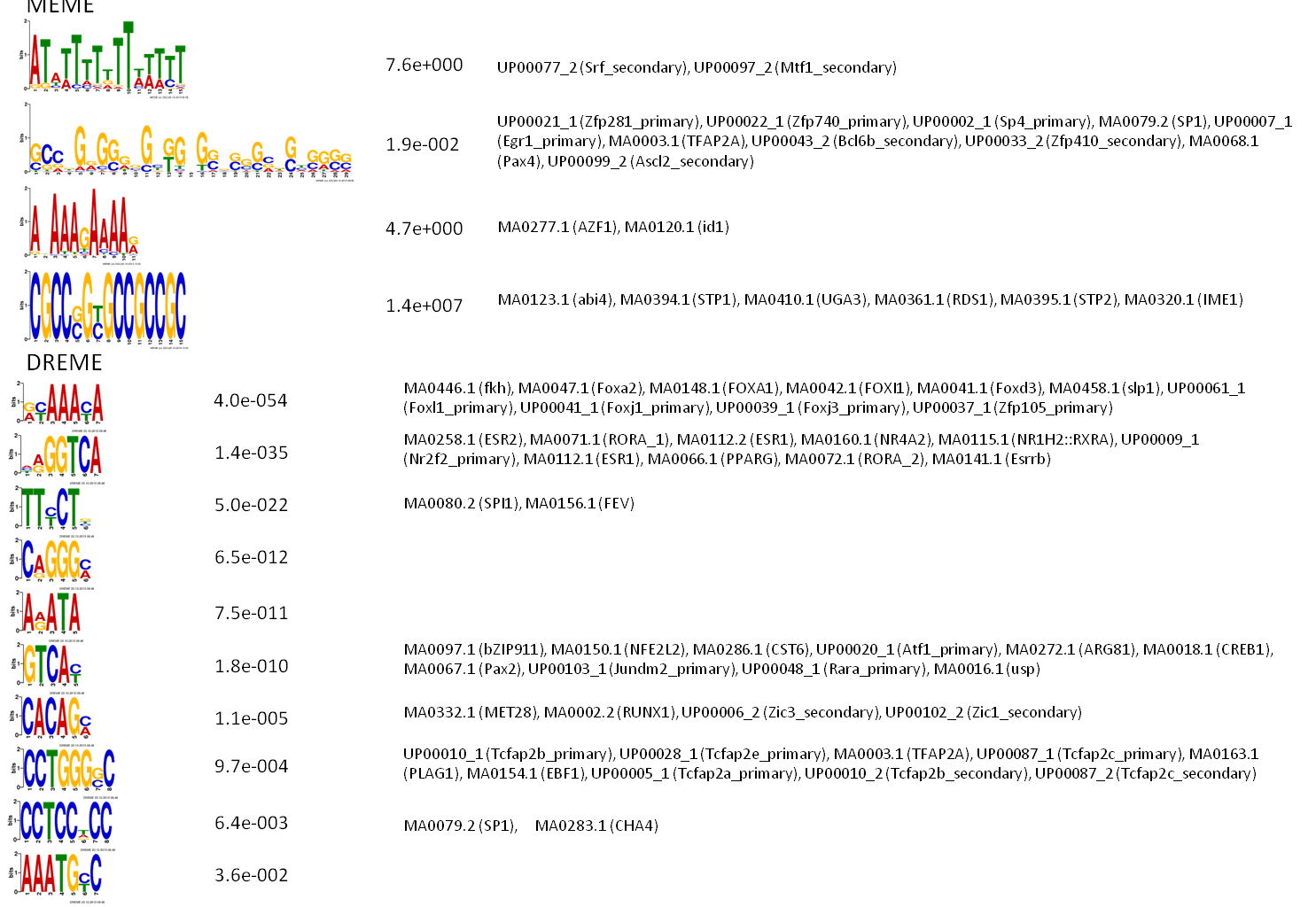

Supplement: Additional file 4: Figure S3 — MEME- and DREME- identified motifs within ESR1 binding sites without classical ESR1 recognition motifs. E-values are shown for each motif along with TOMTOM similarity to known motifs (JASPAR (upper case) and uniprobe mouse (lower case) with E-value <10). [file 1755-8794-6-45-S4.tiff]
